# A novel extrachromosomal circular DNA related genes signature for overall survival prediction in patients with ovarian cancer

**DOI:** 10.1186/s12920-023-01576-x

**Published:** 2023-06-19

**Authors:** Ying Zhang, Kexian Dong, Xueyuan Jia, Shuomeng Du, Dong Wang, Liqiang Wang, Han Qu, Shihao Zhu, Yang Wang, Zhao Wang, Shuopeng Zhang, Wenjing Sun, Songbin Fu

**Affiliations:** 1 Laboratory of Medical Genetics, Harbin Medical University, Harbin, 150081 China; 2 Key Laboratory of Preservation of Human Genetic Resources and Disease Control in China (Harbin Medical University), Ministry of Education, Harbin, 150081 China; 3 Scientific Research Centre, The Second Affiliated Hospital of Harbin Medical University, Harbin, 150081 China; 4 Department of Gynecology, Harbin Medical University Cancer Hospital, Harbin, 150081 China

**Keywords:** Ovarian cancer, eccDNA, TCGA, Prognostic model, Immune infiltration

## Abstract

**Objective:**

Ovarian cancer (OV) has a high mortality rate all over the world, and extrachromosomal circular DNA (eccDNA) plays a key role in carcinogenesis. We wish to study more about the molecular structure of eccDNA in the UACC-1598–4 cell line and how its genes are associated with ovarian cancer prognosis.

**Methods:**

We sequenced and annotated the eccDNA by Circle_seq of the OV cell line UACC-1598–4. To acquire the amplified genes of OV on eccDNA, the annotated eccDNA genes were intersected with the overexpression genes of OV in TCGA. Univariate Cox regression was used to find the genes on eccDNA that were linked to OV prognosis. The least absolute shrinkage and selection operator (LASSO) and cox regression models were used to create the OV prognostic model, as well as the receiver operating characteristic curve (ROC) curve and nomogram of the prediction model. By applying the median value of the risk score, the samples were separated into high-risk and low-risk groups, and the differences in immune infiltration between the two groups were examined using ssGSEA.

**Results:**

EccDNA in UACC-1598–4 has a length of 0-2000 bp, and some of them include the whole genes or gene fragments. These eccDNA originated from various parts of chromosomes, especially enriched in repeatmasker, introns, and coding regions. They were annotated with 2188 genes by Circle_seq. Notably, the TCGA database revealed that a total of 198 of these eccDNA genes were overexpressed in OV (*p* < 0.05). They were mostly enriched in pathways associated with cell adhesion, ECM receptors, and actin cytoskeleton. Univariate Cox analysis showed 13 genes associated with OV prognosis. LASSO and Cox regression analysis were used to create a risk model based on remained 9 genes. In both the training (TCGA database) and validation (International Cancer Genome Consortium, ICGC) cohorts, a 9-gene signature could successfully discriminate high-risk individuals (all *p* < 0.01). Immune infiltration differed significantly between the high-risk and low-risk groups. The model’s area under the ROC curve was 0.67, and a nomograph was created to assist clinician.

**Conclusion:**

EccDNA is found in UACC-1598–4, and part of its genes linked to OV prognosis. Patients with OV may be efficiently evaluated using a prognostic model based on eccDNA genes, including SLC7A1, NTN1, ADORA1, PADI2, SULT2B1, LINC00665, CILP2, EFNA5, TOMM.

**Supplementary Information:**

The online version contains supplementary material available at 10.1186/s12920-023-01576-x.

## Introduction

OV is the second most frequent malignancy in women’s health and the leading cause of mortality from gynecological cancers [1–6]. About 70% of patients with OV are not detected until late stages because of a lack of early symptoms, indicators, and effective screening tools [7, 8]. The majority of patients are unable to have surgery due to metastases [1, 9]. They can only get palliative therapies like radiation and chemical therapy [10, 11]. Advanced patients have a 5-year survival rate of just 20% [12]. The molecular mechanism behind the occurrence and progression of OV is yet unknown, which makes early detection of the disease challenging [13]. As a result, a thorough knowledge of the biological and molecular mechanisms that contribute to the advancement of OV, as well as the identification of crucial variables in the incidence and progression of OV, is critical for further research into efficient early detection and treatment techniques for OV.

The hypothesis of “mutation and carcinogenesis” is now the most commonly recognized theory on the process of malignant transformation and progression of tumor cells [8, 11, 14]. Previous research has established a functional association between copy number variations (CNVs) and carcinogenesis in human malignancies [15]. eccDNA is a small circular DNA that is found outside of the chromosome and may self-replicate independent from chromosome [16–18]. EccDNA is formed by a variety of chromosomal events, and its amplification can directly increase the copy number of oncogenes, speeding up the generation and development of tumors [18]. According to Paulsen’s research, eccDNA can be transcribed and affect gene expression in cells [19]. Kim discovered that in glioblastoma, the epidermal growth factor receptor (EGFR) gene was frequently mutated to EGFRvIII, which could offer tumor cells an advantage in terms of proliferation. Furthermore, EGFR VIII was mostly amplified on eccDNA, making tumor cells more vulnerable to TKI treatment [20]. In Wu’s study, GBM39 circular ecDNA was analyzed in combination with TCGA database and explored its potential value in tumor therapy [21]. With the development of sequencing technology and improvement of experimental methods, Moller et al. proposed an experimental method for the extraction, enrichment and purification of eccDNA in 2018 [17]. Its core idea is to remove linear DNA using an ATP-dependent exonuclease, leave eccDNA alone, circularly amplify it using the rolling circle approach, and sequence it using second-generation sequencing. Gene annotation was done using Circle-map software. We believe that these fragments on eccDNA do not appear accidentally based on previous research [17]. eccDNA can be transcribed into mRNA and play important roles in tumor. To explain these eccDNA functions, we tried to use the TCGA database to determine whether these genes play an important role in the development of OV. The overexpression eccDNA in OV will be enriched and analyzed by bioinformatics method. The risk model will be constructed by LASSO analysis and Cox regression model to obtain the prognostic model of OV and compare the effects of gene tags related to eccDNA on the occurrence of OV.

This is the first study to use eccDNA gene to build a prognostic model in OV, even in all tumors. Our research will provide a more comprehensive landscape of eccDNA in UACC-1598–4, as well as valuable information about the clinical roles of eccDNA in malignancies. It will provide a novel idea to evaluating eccDNA roles in OV prognosis.

## Materials and methods

### Cell lines and culture conditions

The ovarian cancer cell line UACC-1598 was a kind gift from Dr. Xin-Yuan Guan (University of Hong Kong). The UACC-1598–4 cell line was a clone of UACC-1598–4 selected for the stable maintenance of a high number of extrachromosome circular DNA. SKOV3, Ovarian cancer cell lines without extrachromosome circular DNA used as controls (Supplementary Fig. 1). UACC-1598–4 and SKOV3 was maintained in Roswell Park Memorial Institute 1640 (RPMI1640) media (GIBCO, Carlsbad, CA, USA) supplemented with 10% fetal bovine serum (FBS). Cells were grown at 37℃ in a humidified atmosphere of 5% CO_2_ and passaged every 2 to 3 days when they grew confluent.

### Extraction and purification of eccDNA

UACC-1598–4 cell line was alkaline treated to separate chromosomal DNA, lipids, and protein from eccDNA by rapid DNA denaturing–renaturing, followed by column chromatography on an ion exchange membrane column (Plasmid Mini AX; A&A Biotechnology), The eccDNA was purified according to the purification method provided in the document, linear and mitochondrial DNA were removed by endonucleases and rolling-circle amplification of eccDNA by Phi29 polymerase reactions (REPLI-g Midi Kit) amplifying DNA at 30 °C for 2 days for Circle-Seq and then the eccDNA was sequenced based on Illumina platform [17].

### EccDNA data analysis

Briefly, Circle-seq data, the data analysis process is as follows: 1. FASTQC software to evaluate the quality of the original data. 2. BWA software to compare the original data to the reference genome. 3. Samtools to process the SAM file to fit the format required by Circle-map. 4. Circle-map to detect eccDNA. 5. Gene annotation to eccDNA, Differential eccDNA analysis and annotated gene function enrichment analysis, Circle-seq data analyses were performed by ZHONGKE SHENGXIN Biotechnology, Beijing, China.

### Data collection and mining of mRNA profiles

We obtained the RNA sequencing (RNA-seq) data of 379 OC patients and the corresponding clinical features from the TCGA database (https://portal.gdc.cancer.gov/repository). The RNA-seq data of 88 normal human ovarian samples were downloaded from the GTEx database (https://xenabrowser.net/datapages/). For further verification, the clinical data and transcriptional patterns were obtained from OV cases in the International Cancer Genome Consortium (ICGC) data-base (https://icgc.org/).

### Identification of amplified genes in OV

GEPIA2 analysis Gene Expression Profiling Interactive Analysis (GEPIA, http://gepia2.cancer-pku.cn, version 2) is an open-access online tool for the interactive exploration of RNA sequencing data of 9736 tumors and 8587 normal samples from the TCGA and the Genotype-Tissue Expression (GTEx) programs [22]. In this study, GEPIA2 was used to obtain the amplified genes Under the condition of log2FC > 1, *p* < 0.05, OV specific amplified genes in TCGA and GTEx databases were obtained.

### Construction of a signature based on eccDNA genes

A univariate Cox regression model was adopted to determine the hazard ratios (HR) of prognosis prediction for eccDNA genes [23]. Univariate or multivariate Cox analysis was employed to determine the prognostic value for the risk signature or clinical features using the ‘forest’ R package. Genes were further screened by LASSO regression analysis, followed by tenfold cross validation using the ‘glmnet’ R package [24]. Nine genes with their regression coefficient (Coef) were selected, the risk score for each patient was calculated through linearly multiplying the expression level with Coef of each gene, according to the following formula: Risk score = Coef gene1 × expression gene1 + Coef gene2 × expression gene2 + · ···· + Coef gene n × expression gene n [25]. Each patient was assigned a risk score based on the formula and divided into either high-risk group or low-risk group according.

### Confirmation of the signature based on eccDNA genes

Subsequently, the receiver operating characteristic (ROC) curves were performed to assess the sensitivity and specificity of survival prediction by the risk signature using the ‘survivalROC’ R package. Univariate and multivariate Cox proportional hazards regression analysis was performed to determine whether the risk score is an independent predictor for prognosis.

### Nomograph drawing

In order to construct a quantitative scoring system for prognostic evaluation of OV samples, the appropriate indicators were selected to construct a nomogram. The construction of nomogram Age, stage, and risk score were used to construct the nomogram together using the “rms” and “survival” packages in R. Calibration curves were drawn to assess the consistency between actual and predicted survival.

### Functional enrichment analysis

The “ClusterProfiler” R package was utilized to conduct Gene Ontology (GO) and Kyoto Encyclopedia of Genes and Genomes (KEGG) [26] analyses based on the eccDNA related OV amplification genes. *P* values were adjusted with the BH method.

### Immune cell infiltration estimation

The ssGSEA method was used to calculate the enrichment levels of immune cell infiltration in ovarian cancer cohorts. The difference in the immune cell infiltration between the low and high-risk group was carried out by Wilcoxon tests, with the *p* value less than 0.05 as statistically significant.

### Statistical analysis

Wilcoxon tests was used to compare Immune infiltration between high-risk group and low-risk group. The OS between different groups was compared by Kaplan–Meier analysis with the log-rank test. All statistical analyses were performed with R software (Version 4.0.0). If not specified above, a *p*-value less than 0.05 was considered statistically significant, and all *p*-values were two-tailed.

### Transwell assay

For the migration assay, approximately 1.5 × 10^4^ cells were placed in 200ul serum-free medium in the upper chamber of the transwell system. For the invasion assays, the upper chamber was covered with matrigel and placed in a 37℃ incubator for 4 h to allow matrigel to solidify. Approximately 1.5 × 10^4^ cells were placed in 200 ul serum-free medium in the upper chamber of the transwell system. 600 mL RPMI 1640 medium containing 10% FBS was placed in the lower chamber as a chemoattractant. After 24 h of incubation, cells in the upper chamber were removed, and the lower chamber was fxed with formaldehyde and stained with crystal violet. The number of cells was counted using Image J software. All experiments were repeated at least thrice.

## Results

### Overview of the overall analysis process

**Fig. 1:**
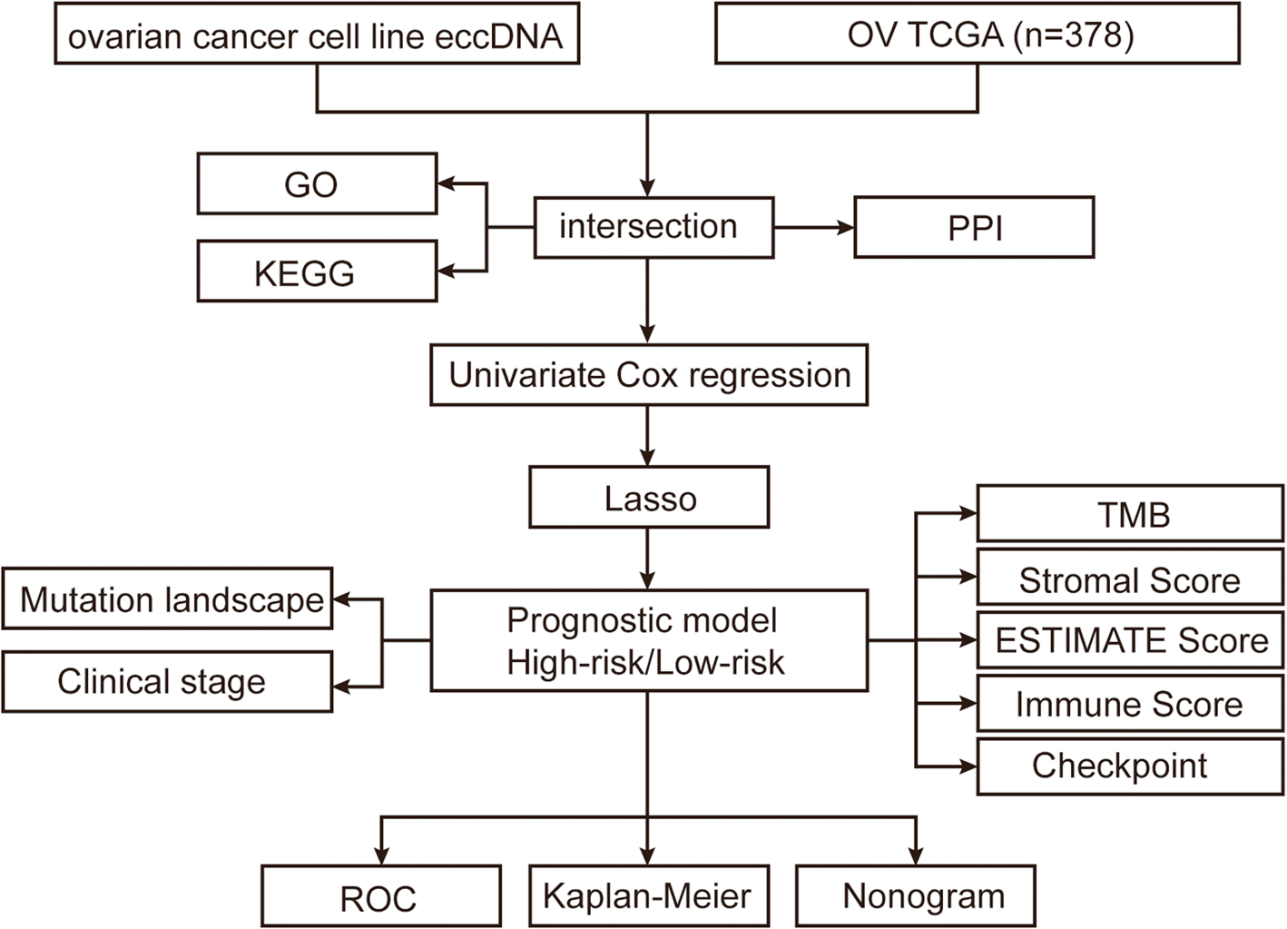
Flow chart of data collection and analysis

To acquire the eccDNA genes overexpression in OV, the genes annotated by eccDNA in UACC-1598–4 intersect with the upregulated genes on OV obtained from the TCGA database. GO and KEGG were utilized to evaluate pathways of these OV related eccDNA genes in order to better understand their mechanism in the occurrence and progression of OV. Univariate Cox regression was used to identify genes associated with OV prognosis, which were then screened using LASSO, a prognostic model was built by LASSO cox analysis, and the model’s efficacy was scientifically validated. Figure 1 depicts the study’s flow chart.

### Landscape of eccDNA characteristics and identification amplified genes on eccDNA in OV

**Fig. 2:**
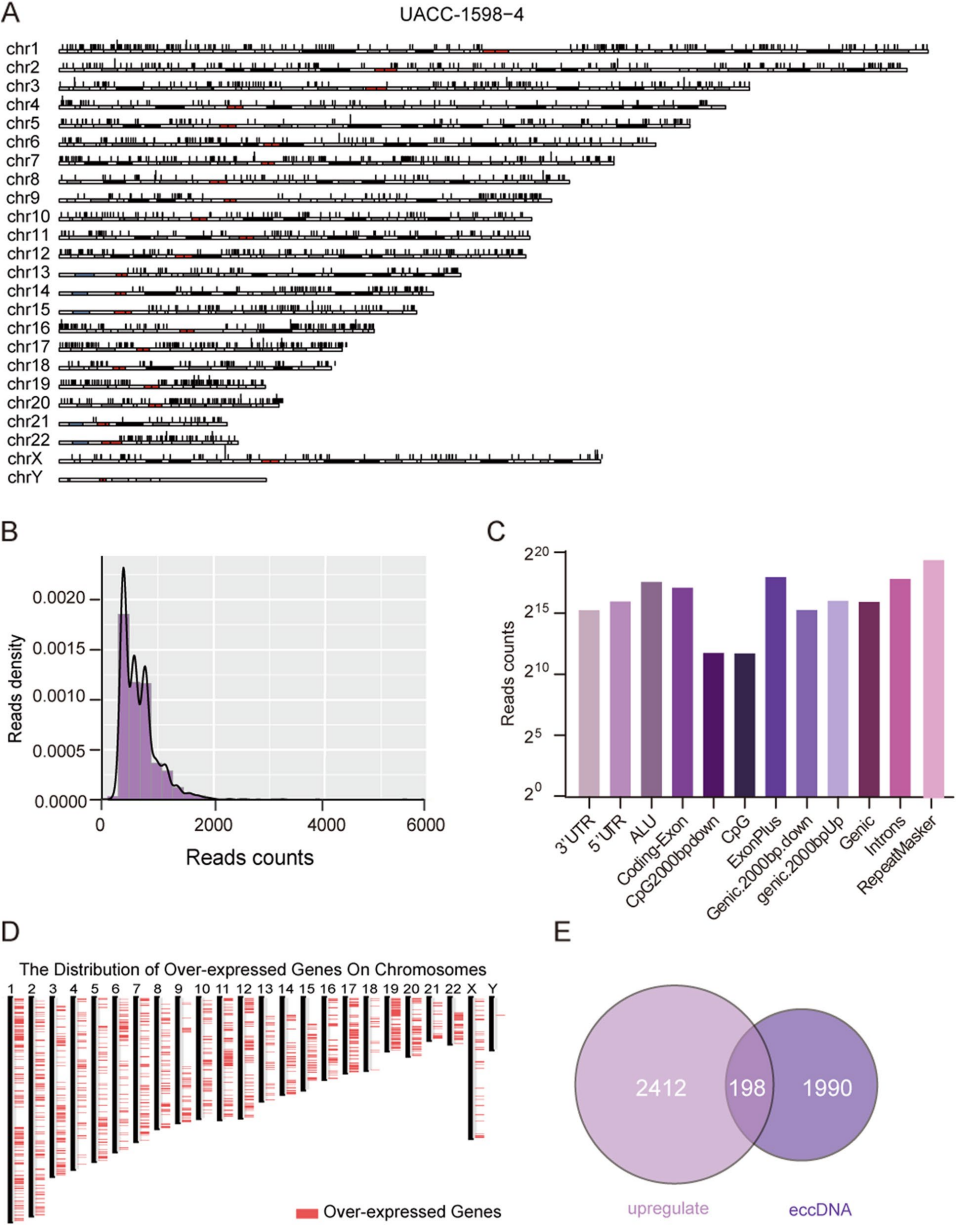
Landscape of overall analysis characteristics of eccDNA. **A** Distribution landscape of eccDNA in chromosome source. **B** Length distribution of eccDNA shows peaks at 200 -600 bases. **C** The sites in the genome that give rise to small eccDNA are enriched relative to random expectation in genic sites, The top three are RepeatMasker, ExonPlus, Introns. **D** The significantly amplified genes on OC were obtained by GEPIA2. **E** Intersection of eccDNA gene and OV amplified gene

The extracted eccDNA genome was sequenced on the Illumina platform, and the Circle_seq method was used to identify and annotate eccDNA. The number of split reads and discordant reads were used to score the possibility of a circle. The top 15 eccDNA score genes were given in Table 1. The positional distribution of eccDNA on the UACC-1598–4 chromosome is shown in Fig. 2A. It can be seen that eccDNA is distributed on all chromosomes. Under strict filtration conditions, a total of 2188 eccDNA genes were annotated. As shown in Fig. 2B, the length ranges from 0 to 2000 bp, with the majority falling between 400 and 600 bp. The source of eccDNA in the genome is depicted in Fig. 2C, the majority of which are repeatmasker areas, but it also can be found in the UTR region, gene coding regions and so on. GEPIA2 (http://gepia2.cancer-pku.cn/#index) was used to get a total of 2610 genes amplified in OV compared with normal samples. Figure 2D shows the distribution of genes amplified on OV in the genome. There are 198 genes intersect with genes on eccDNA as shown in Fig. 2E.

**Table 1:** The top 15 eccDNA location and the annotated genes

Chr	Start	End	Discordant reads	Split reads	Gene name
chr19	39932344	39932930	18	10	FCGBP
chr5	3484327	3484685	8	10	LINC01019
chr5	83505278	83506203	6	10	VCAN
chr8	134602587	134602967	6	10	ZFAT
chr22	43887811	43888202	7	9	PNPLA5
chr17	50512702	50513101	8	10	MYCBPAP
chr2	68645344	68645830	8	10	PROKR1
chr10	55471553	55472626	9	10	PCDH15
chr17	33850812	33851395	14	10	ASIC2
chr7	50540627	50541378	10	10	DDC-AS1, DDC
chr6	90064028	90064826	6	9	BACH2
chr16	85076748	85077346	5	10	KIAA0513
chr16	75635660	75636289	9	10	KARS1
chr2	233642951	233643854	8	9	UGT1A10, UGT1A8

### Protein interaction (PPI) analysis and enrichment of eccDNA related OV amplified genes

**Fig. 3:**
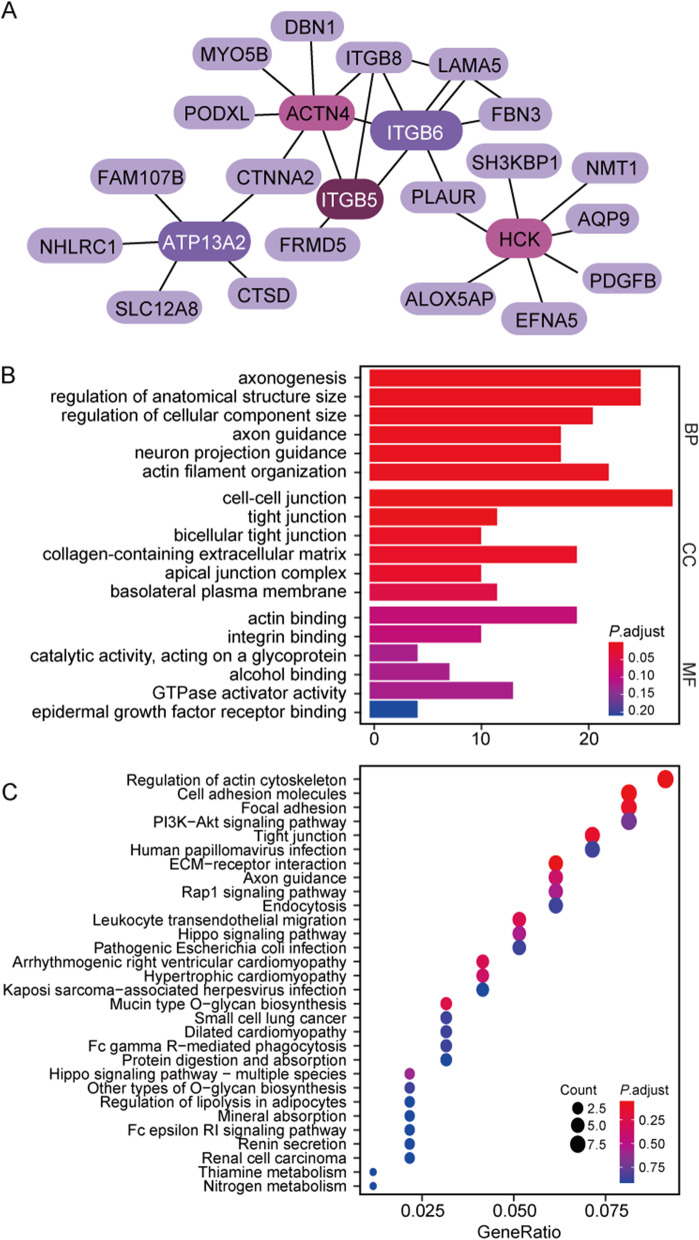
Protein interaction (PPI) analysis and enrichment of eccDNA related OV amplified genes. **A** The string database shows 198 protein interactions (PPI). **B** GO database pathway enrichment analysis results. **C** KEGG database pathway enrichment analysis results

To further explore the interactions of 198 genes, we conducted a PPI analysis. To make it easier to display, Fig. 3A only shows the genes in the top5 nodes ranked by degree calculated by the Cytoscape plug-in cytohubba. It can be seen that ACTN4, HCK, ITGB5, ITGB6 and ATP13A2 genes interact with multiple proteins. As shown in Fig. 3B-C, 198 genes were analyzed for GO and KEGG enrichment. It’s mainly enriched in the regulation of cell adhesion, ECM receptors, the actin cytoskeleon pathway and so on. This suggests that these genes may have a role in the onset and progression of OV via modulating the cell–cell junction or the dynamic network pathway. We selected ovarian cancer cell lines SKOV3 that do not contain extracellular circular DNA for experimental analysis (Supplementary Fig. 1). Our experimental results showed that the expression of marker vimentin in mesenchymal cells was significantly increased in UACC-1598–4 cells containing eccDNA(Supplementary Fig. 2). In addition, we supplemented the cell migration experiment and found that the migration and invasion ability of UACC-1598–4 cell line was significantly enhanced. (Supplementary Fig. 3). This echoes the enrichment results of the ECM receptor pathway and the cell adhesion pathway.

### Univariate Cox regression analysis of eccDNA amplified genes in OV associated with prognosis

**Fig. 4:**
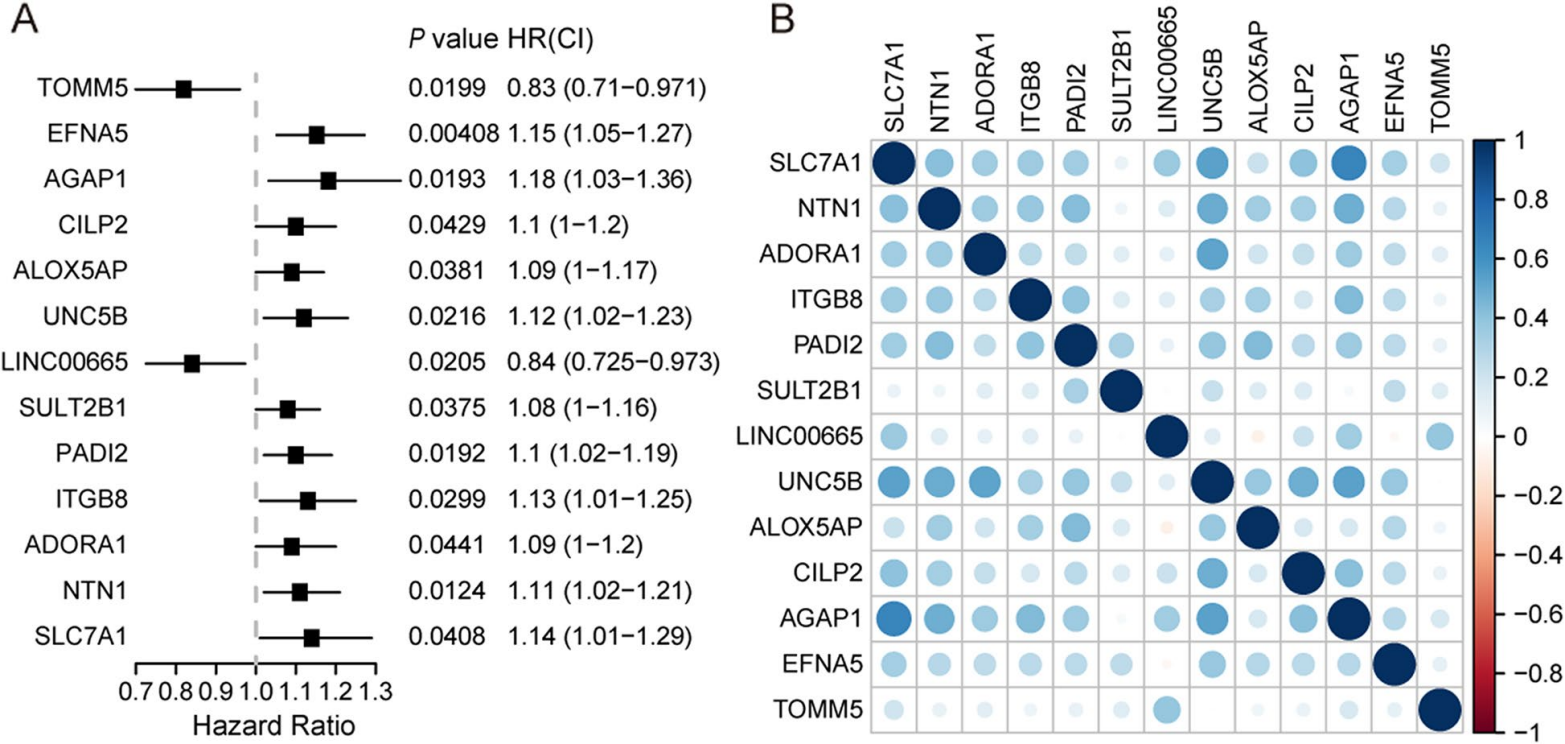
Univariate Cox regression analysis of amplified genes in OV associated with prognosis. **A** Hazard ratio and *P*-value of constituents involved in Univariate Cox regression and some parameters of the eccDNA signature. The left side of the dotted line represents HR < 1, which is a protective factor, and the right side represents HR > 1, which is a risk factor. **B** Correlation matrix of hub genes implicated in eccDNA

The survival-related genes were first screened using univariate Cox regression analysis. As shown in Fig. 4A, the 13 genes (SLC7A1, NTN1, ADORA1, ITGB8, PADI2, SULT2B1, LINC00665, UNC5B, ALOX5AP, CILP2, AGAP1, EFNA5, TOMM5) that met the criteria of *P* < 0.05 were retained for further analysis. There are 11 potentially hazardous genes and two potentially protective genes among them. Except for TOMM5 and LIN00665, all other genes had HR values more than 1, indicating that the majority of them are OV risk genes. The correlation coefficient heatmap is shown in Fig. 4B. It can be seen that there is correlation among the 13 genes, specifically mentioned, there is an obvious negative correlation between AGAP1 and SCL7A1 (*p* < 0.05, *r* =—0.65), implying that there may be potential interactions between these genes.

### Further screen genes by LASSO and correlation study

**Fig. 5:**
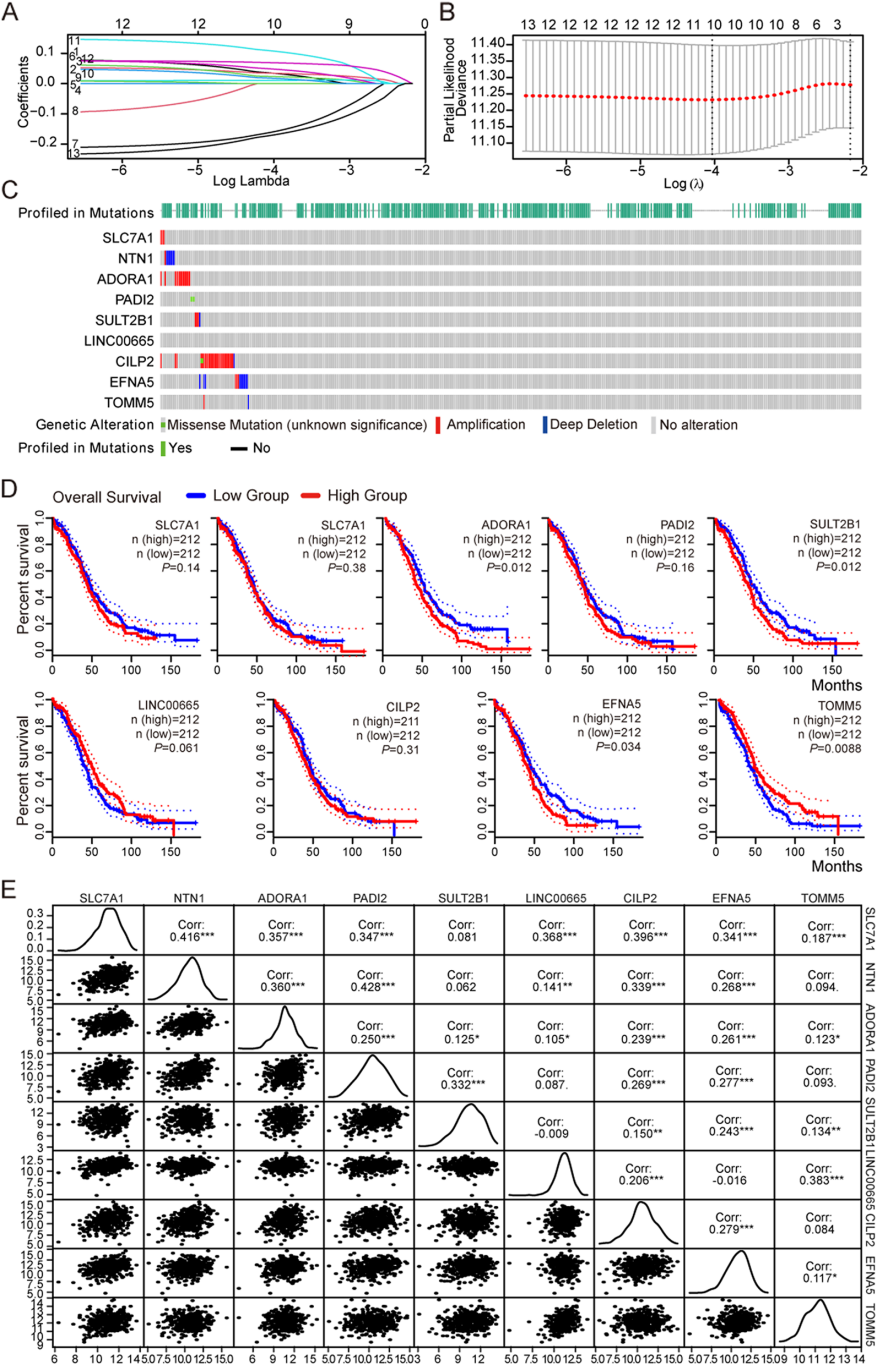
Further screen genes by LASSO and correlation study. **A** LASSO coefficient profiles of the 13 genes in TCGA-OV. Different colors represent different variables (genes). **B** λ selection diagram. The two dotted lines indicated two particular values of λ. The left side was λmin and the right side was λ1se. The λmin was selected to build the model for accuracy in our study. **C** Genetic alteration of the 9 genes in the TCGA-OV cohort (cBioPortal). **D** The prognosis of 9 genes obtained from GEPIA2 database. **E** Correlation analysis among 9 genes by spearman correlation

For a more precise prediction of OV prognosis by eccDNA genes, the cox regression algorithm penalized by LASSO was utilized. The λ selection diagram is shown in Fig. 5A-B. λ between λmin and λ_1_se were considered appropriate. The model constructed by λ_1_se was the simplest, that was, it used a small number of genes, while λmin had a higher accuracy rate and used a larger number of genes. The λmin was selected to build the model for accuracy in our study. Patients in the training and validation cohorts were divided into low- or high-risk subgroups based on the median of risk scores [24]. After cross validation, 9 genes, including SLC7A1, NTN1, ADORA1, PADI2, SULT2B1, LINC00665, CILP2, EFNA5, TOMM5 were chosen for calculation of a risk signature. The genetic alterations of 9 eccDNA were analyzed by cBioPortal database as shown in Fig. 5C. Most of genes were significantly amplified except LINC00665, among which PADI2 and CILP2 had missense mutation. In addition, Kaplan Meier survival curve for these 9 genes was carried out, as shown in Fig. 5D, among which ADORA1 (*p* = 0.012), EFNA5 (*p* = 0.034), SULT2B1 (*p* = 0.011) had significant correlation with prognosis, they were related poor prognosis in OV. TOMM5 was significant correlation with prognosis (*p* = 0.0087) but it was a protective factor. Significant correlation with each other except LINC00665, which was not correlation with PADI2, SULT2B1 (*p* > 0.05), TOMM5, which was not correlation with NTN1, PADI2, CILP2 (*p* > 0.05). This suggests that most eccDNA genes may interact in the onset of OV, some of these genes may have synergistic effects, while others may function independently (Fig. 5E).

### Identification of a risk signature comprising of 9 eccDNA genes in OV

**Fig. 6:**
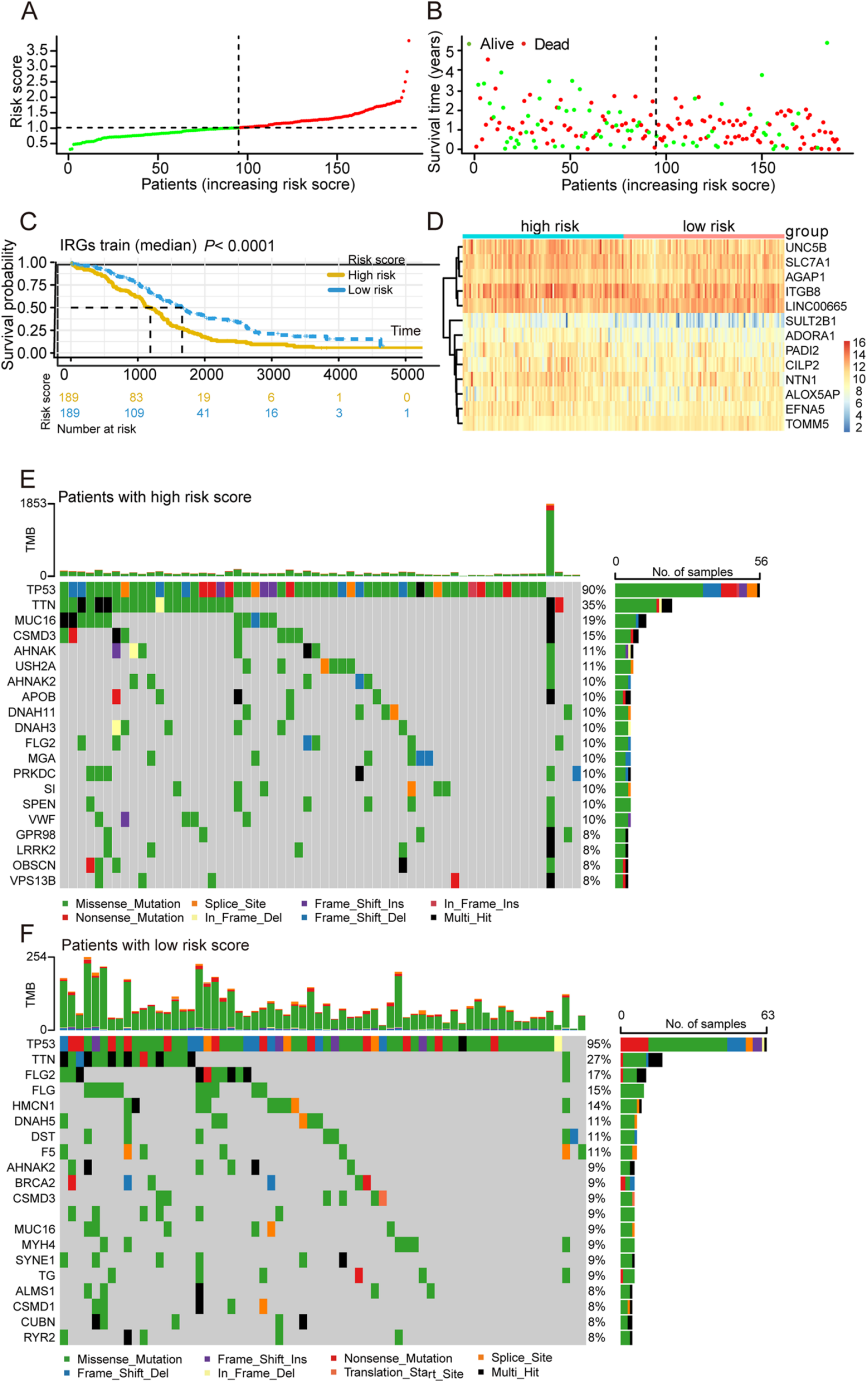
Identification of a risk signature comprising of 9 eccDNA genes in OV. **A** Distribution of patients in the TCGA cohort based on the median risk score. **B** The survival status for each patient (low-risk population: on the left side of the dotted line; high-risk population: on the right side of the dotted line). **C** Kaplan–Meier survival curve between high and low-risk groups. Red lines represent high risk patients, while blue lines represent low risk patients. **D** The heatmap of the expression profiles of 13 prognostic related genes signature. **E** Representative diagram of mutation landscape from the high-risk OV cohort. **F** Representative diagram of mutation landscape from the low-risk OV cohort

Each patient’s risk score was determined using the gene expression values of nine genes and the LASSO regression coefficient. (0.10781*SLC7A1) + (0.06002*NTN1) + (0.03454*ADORA1) + (0.01211*PADI2) + (0.06412*SULT2B1) + (-0.18898*LINC00665) + (0.03455*CILP2) + (0.07681*EFNA5) + (-0.21118*TOMM5) The samples are separated into high-risk and low-risk groups based on the median value of the risk score (Fig. 6A-B). The prognosis of the samples in the low-risk group is considerably better than that of the samples in the high-risk group (*p* < 0.05), as shown in Fig. 6C. Figure 6D shows the expression of 13 prognosis-related genes in high-risk and low-risk groups. To better understand the differences between the two groups. The mutation landscape of genes in high and low risk groups was analyzed, Fig. 6E shows the mutation landscape among high-risk group samples, it was found that TP53 exhibited the highest mutation frequency followed by TTN, MUC16, CSMD3, AHNAK2, and USH2A, etc. Figure 6F shows the mutation landscape among Low-risk group samples, 17 had mutations in these genes, with frequency of 13%. It was found that TP53 exhibited the highest mutation frequency followed by TTN, FLG2, FLG, HMCN1 and DNAH5, etc.

### Comparison of immune infiltration and immune checkpoints between high and low-risk groups

**Fig. 7:**
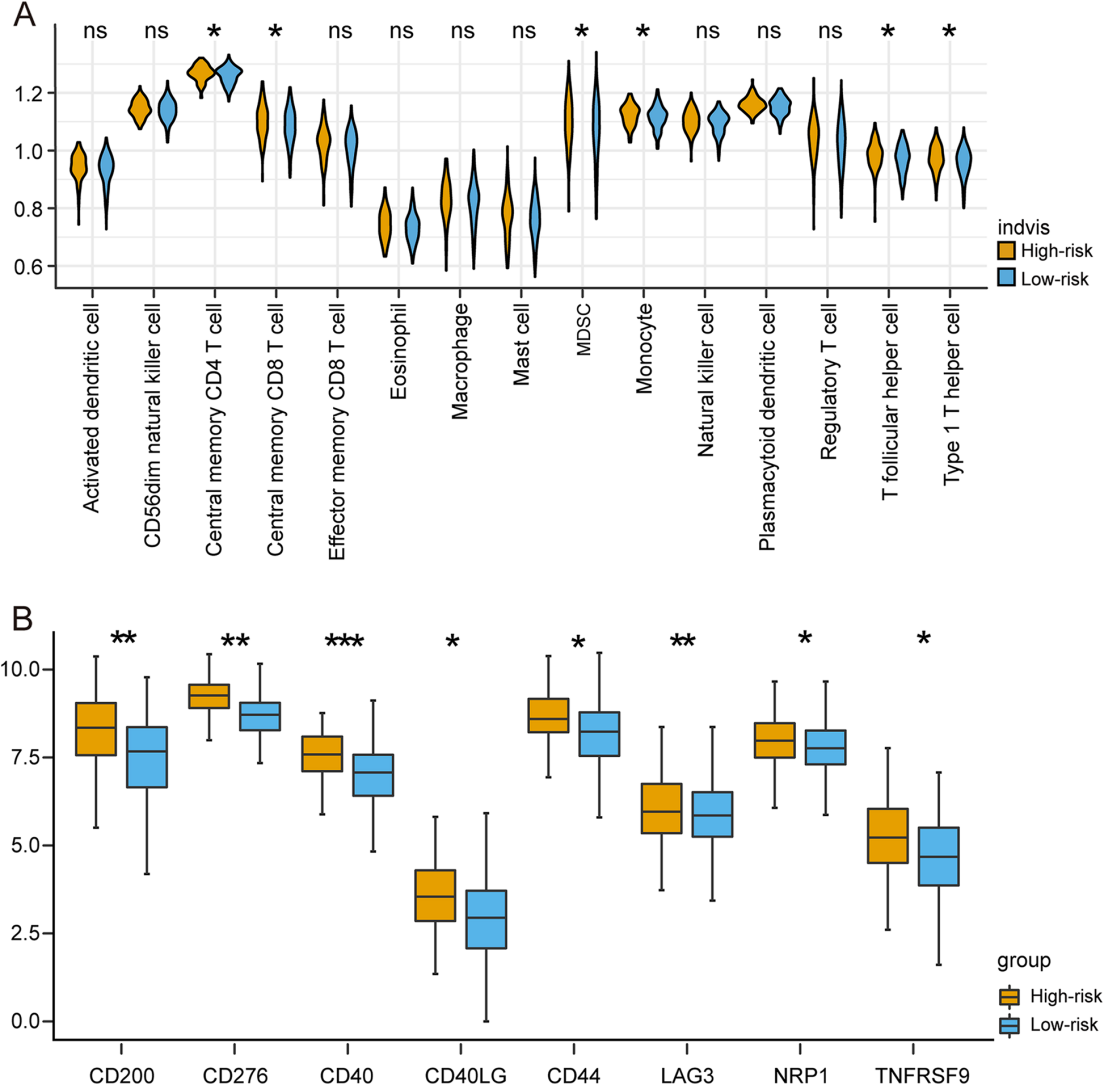
Comparison of immune infiltration and immune checkpoint between high and low-risk group. **A** The expression levels of 28 immunity cells were analyzed by ssGSEA. **B** Among the 29 immune checkpoint genes, 6 genes were differentially expressed between high and low risk group, *P* values were showed as: *P < 0.05, **P < 0.01, ***P < 0.001

Immune infiltration was used to get new understanding into the biological role of each of the risk groups. The violin plots depicted the relative enrichment levels of 28 immune cells (Fig. 7A), Central memory CD4 T cell, Central memory CD8 T cell, MDSC, and Monocyte, T follicular helper cell, Type 1 T helper cell have higher levels in the high-risk category. We also looked at the differences between high-risk and low-risk groups in 29 immunological checkpoints. Only significant different checkpoints between the two groups are shown in Fig. 7B. CD200, CD40, CD44, LAG3, NRP1, CD276, CD40LG, NRP1, and TNFRSF9 are shown to be significantly overexpression in high-risk groups (*p* < 0.05). These findings suggest that the high-risk group, as characterized by the eccDNA profile, had more immune infiltration.

### ROC evaluating diagnostic effectiveness and building a predictive nomogram

**Fig. 8:**
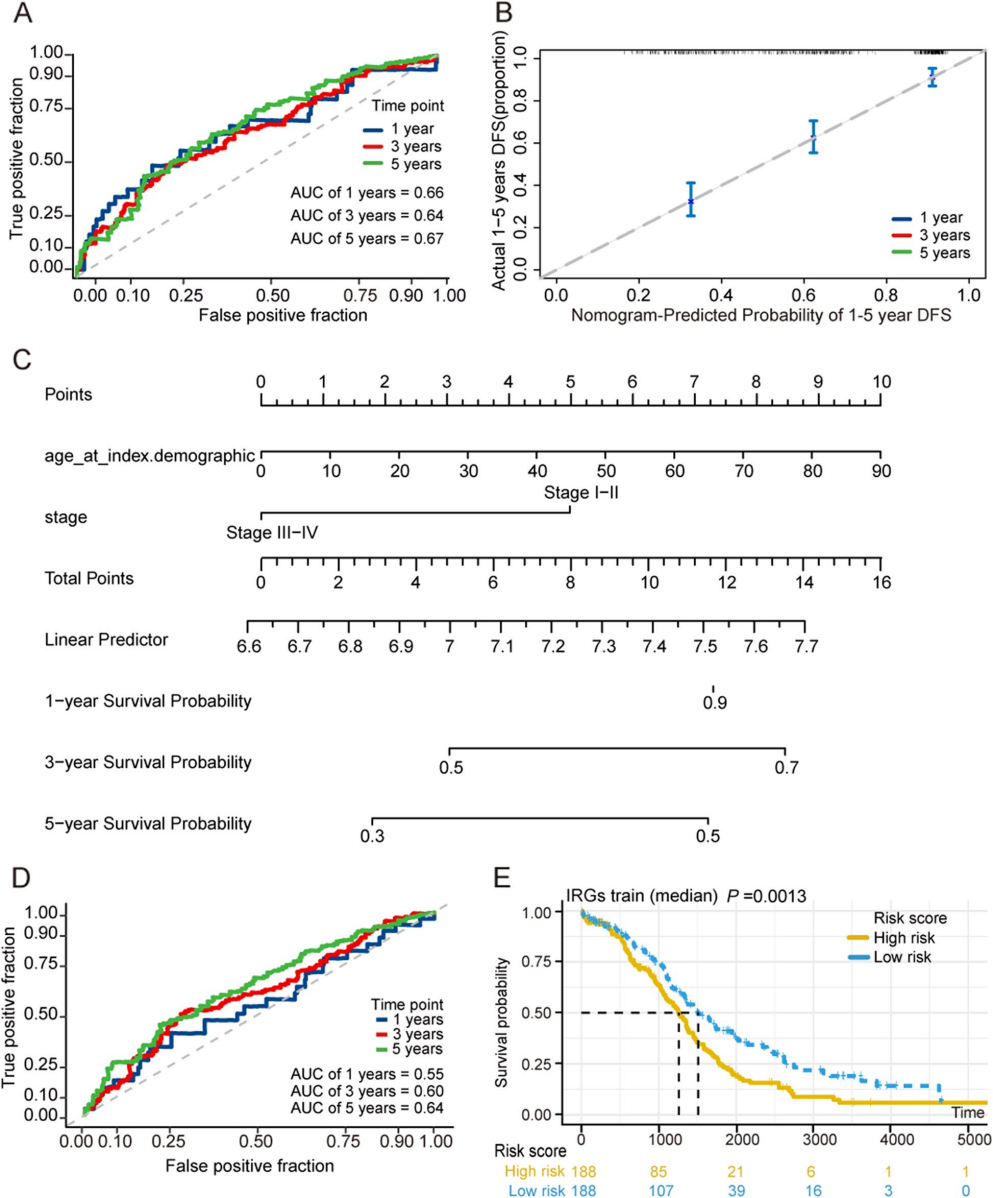
ROC evaluating diagnostic effectiveness and building a predictive nomogram. **A** ROC curve was plotted for 1-, 3- and 5-years overall survival in the TCGA group. **B** Nomogram to predict the 1-years, 3-years and 5-years overall survival of OV patients. **C** Calibration curve for the overall survival nomogram model in test group. **D** ROC curve was plotted for 1-, 3- and 5-years overall survival in the ICGC group. **E** Kaplan–Meier survival curve results in ICGC groups

ROC curves for one-year, three-year, and five-year survival periods were produced to assess the prognostic efficacy of the prognostic model. The area under the curve (AUC) in one year was 0.66, three years was 0.64, and five years was 0.67, as illustrated in Fig. 8A. The nomogram was then created based on the risk score and three clinicopathological risk indicators to give clinicians with a quantitative technique to estimate the probability of cancer development. Each factor’s score is proportionate to how much of a risk it poses to survival. The calibration curve indicator is accurate (Fig. 8B-C). We also looked at how our model performed in different data sets. ROC curves were drawn using OV data on ICGC, and it can be seen that its diagnostic efficacy is almost same to that of TCGA (Fig. 8D). Furthermore, in the ICGC data set, the high-risk group’s survival was significantly worse than the low-risk group’s (Fig. 8E), GSE72094 dataset also confirming the model’s reliability ((Supplementary Fig. 4).

## Discussion

We demonstrated that the application of Circle-Seq pipeline can identify eccDNA in UACC-1598–4 cell line. The diversity of eccDNA found in this study supports the idea that eccDNA can come from any section of the human genome. However, there are specific hotspots in eccDNA production in the human genome: RepeatMasker, ExonPlus, and Introns had a greater frequency of circularization and formation of eccDNA, which is consistent with prior human germline and yeast data [27, 28]. The length distribution of eccDNA ranges from 0-2000 bp, with a peak of 400-600 bp. Kumar’s research found high levels of eccDNA in tumor mice and human blood, with lengths ranging from 0–2000 bp and an enriched region between 200 and 600 bp. [29]. We speculate that eccDNA can be released from tissue into the blood due to similar length. Pankaj Kumar also used the ATAC-seq combined Circle finder method to identify the eccDNA of the C4-2B (prostate cancer) and OVCAR8 (ovarian cancer) cell lines, finding that 68% of the eccDNA in C4-2B and 37% in OVCAR8 are smaller than 1 kb, and 32% of the eccDNA in C4-2B and 63% in OVCAR8 are larger than 1 kb. The eccDNA in their study was derived from all chromosomes, which was identical to our UACC-1598–4 cell line’s results [30]. In human tumors, in addition to eccDNA, there also exist large fragments of double minutes (DMs), up to 330 kb with proto-oncogenes [31]. Actually, we also found some long-distance breakpoints (> 3 kb), but their credibility is not high according to the circle-map software. It may be because the number of reads on both sides of the breakpoint is not much different from the contig. Low abundance and Phi29 amplification step in the Circle-Seq method is biased for small and more abundant eccDNAs. Moreover, Circle-seq pipeline does not distinguish between eccDNA derived from a single DNA or several DNA fragments. As a result, we cannot exclude that some detected eccDNAs resembled complex structures [17].

We suspect 198 of these genes may be involved in the development of OV. They were enriched in the cell adhesion molecules and ECM-receptor interaction pathway. These pathways are related to the structural homeostasis and junction of cells Numerous studies have shed light on the connections between malignant transformation, metastasis, and cellular adhesion pathways [32–34]. Cell adhesion molecules, for example, can affect single-cell motility and invasion, which are important in many cancer processes [35]. According to Bao’s research, the ECM-receptor interaction signal pathway is a critical signaling route implicated in the development of breast cancer [36]. As a result, we hypothesize that eccDNA genes regulate tumor pathways via influencing cell adhesion molecules and the ECM-receptor interaction pathway, which will be confirmed in future research through studies. In a study of 198 genes, Univariate Cox regression revealed 13 genes linked to ovarian cancer prognosis. Some of 13 genes, such as AGAP1 and SCL7A1, have a high expression correlation, indicating that the circular structure of eccDNA reduced their distance and synchronized their expression. All 9 genes screened by LASSO regression in our study have been shown to play a role in different tumors from previous studies. SLC7A1 (solute carrier family 7 member 1), for example, could be a SPOP substrate and influence cell phenotypic via regulating arginine metabolism, as well as regulate the hepatoblastoma process [37]. NTN1 (netrin-1) has been proved progressed in ovarian cancer and have the potential for the development of new diagnosis and treatment strategies for ovarian cancer [38–40]. ADORA1 may modulate OIN1-mediated apoptosis in ovarian cancer, making it a possible molecular target for ovarian cancer treatment [41]. ADORA1 may modulate OIN1-mediated apoptosis in ovarian cancer, making it a possible molecular target for ovarian cancer treatment [42]. SULT2B1 expression was diminished by downregulating c-MYC, thereby restraining glycolytic metabolism to inhibit colon cancer cell proliferation and chemoresistance under condition of knockdown of OLR1 [43]. LINC00665 is an oncogenic, Cong et al. had proved LINC00665–miR98–AKR1B10 axis may serve as potential diagnostic biomarkers in LUAD tumorigenesis. CILP2 was identified of an energy metabolism‑related gene signature in ovarian cancer prognosis [44]. EFNA5 is an unfavorable factor in high-grade serous ovarian cancer because it is a non-canonical Eph-receptor ligand [45]. The current main research on TOMM5 focuses on metabolism, and its involvement in tumors has not been reported [46, 47]. This suggests that the majority of those genes have an essential function in tumors; some of those roles have been demonstrated in ovarian cancer alone, but the use of a combination of these genes to diagnose ovarian cancer has not been reported.

The median risk score of samples was used to categorize samples into high-risk and low-risk groups. The Kaplan–Meier curve illustrated that the high-risk and low-risk groups have significantly different survival times (*p* < 0.05). We established nomograms in combination with clinical information and age of onset of patients. It could assist clinicians treat patients with OV by predicting specific death risks. Our study established a good prognosis model in OV for the first time from the perspective of eccDNA, which enriched the understanding of eccDNA.

The relationship between tumor and immunity has gotten a lot of interest as tumor immunotherapy has progressed [48–50]. The number of immune cells infiltrating a tumor is linked to tumor growth, progression, and patient outcome, and has been a hot topic in recent years [51]. The immunostimulant effect of eccDNAs was discovered in Wang’s study, as well as their pathway and potential clinical implications in immune response [52]. Our study provides more specific details of immunomodulation involving eccDNA in OV cell lines. Between the high and low-risk groups, there was a significant difference in central memory CD4 T cells, central memory CD8 T cells, MDSC and Monocyte, T follicular helper cell, Type 1 T helper cell, and certain immunological checkpoints (CD200, CD40, CD44, LAG3, NRP1, CD276, CD40LG, NRP1, and TNFRSF9). According to the findings, the risk model produced by eccDNA genes may affect the prognosis of OV patients by regulated some immune cells or immune checkpoints. High immune infiltration in the high-risk group partly reflected the higher malignancy of the patients and the worse effect of various treatments, implying that our eccDNA signature could distinguish not only patients’ survival prognosis but also their immune cell infiltration levels. This should be discussed further in conjunction with experimental analysis.

For the first time, we isolated and purified eccDNA from an OV cell line and examined its function using the TCGA and GTEx database. Our study showed the landscape of eccDNA in UACC-1598–4 and its roles in the onset and progression of OV. It also gave clinicians useful information when assessing the prognosis of OV patients. However, there were some limitations to this study that need to be addressed. For example, the biological roles of the 9 eccDNA genes should be confirmed in wet experiment, especially in terms of their relationship to immunological infiltration. To make the eccDNA sequencing results more believable, the sample size must be increased. In addition, only one OV cell line was sequenced in this study, which cannot fully and accurately represent all OV patients. The eccDNA gene prognostic risk model should be validated in our clinical center to further determine the diagnostic effectiveness of the model. However, due to insufficient sample size and insufficient follow-up time, it has not yet been completed. Therefore, we will include more kinds of cancer cell lines in future research.

## Supplementary Information

**Additional file 1: Supplementary 1.** Karyotyping analysis reveals the presence of extrachromosomal DNA. **Supplementary 2.** The expression of Vimentinin UACC-1598-4. **Supplementary 3.** Migration and invasion of UACC-1598-4 and SKOV3. **Supplementary 4.** Validation of model prediction effectiveness in GEO dataset GSE72094.

**Additional file 2.**

**Additional file 3.**

## Data Availability

The original contributions presented in the study are included in the article/Supplementary Material. We have uploaded the original sequencing data to the SRA database (PRJNA793615: Circular DNA elements sequencing in UACC-1598–4 cell line).
