# Supplementary material for: A novel extrachromosomal circular DNA related genes signature for overall survival prediction in patients with ovarian cancer
Source: BMC Med Genomics. 2023 Jun 19;16:140. doi: 10.1186/s12920-023-01576-x (PMC10278296; doi:10.1186/s12920-023-01576-x)
Supplement: Supplementary file 1 — Additional file 1: Supplementary 1. Karyotyping analysis reveals the presence of extrachromosomal DNA. Supplementary 2. The expression of Vimentinin UACC-1598-4. Supplementary 3. Migration and invasion of UACC-1598-4 and SKOV3. Supplementary 4. Validation of model prediction effectiveness in GEO dataset GSE72094. [file 12920_2023_1576_MOESM1_ESM.pdf]

Supplementary 1: Karyotyping analysis reveals the presence of extrachromosomal DNA

A

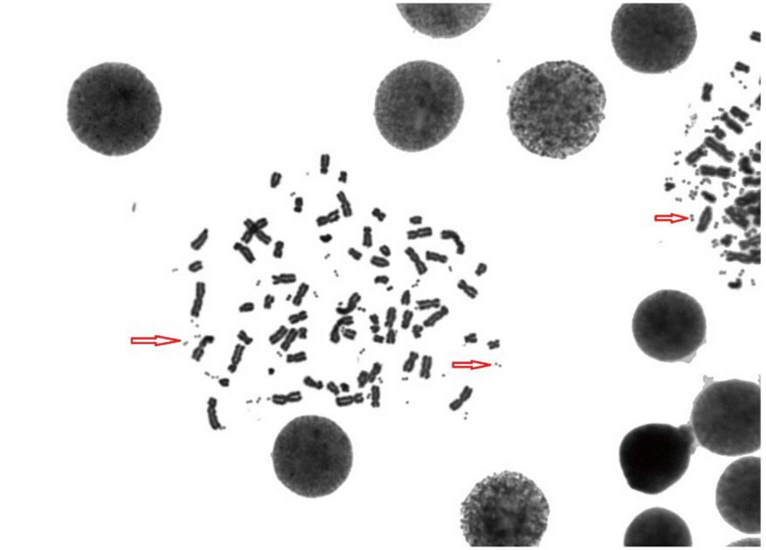

B

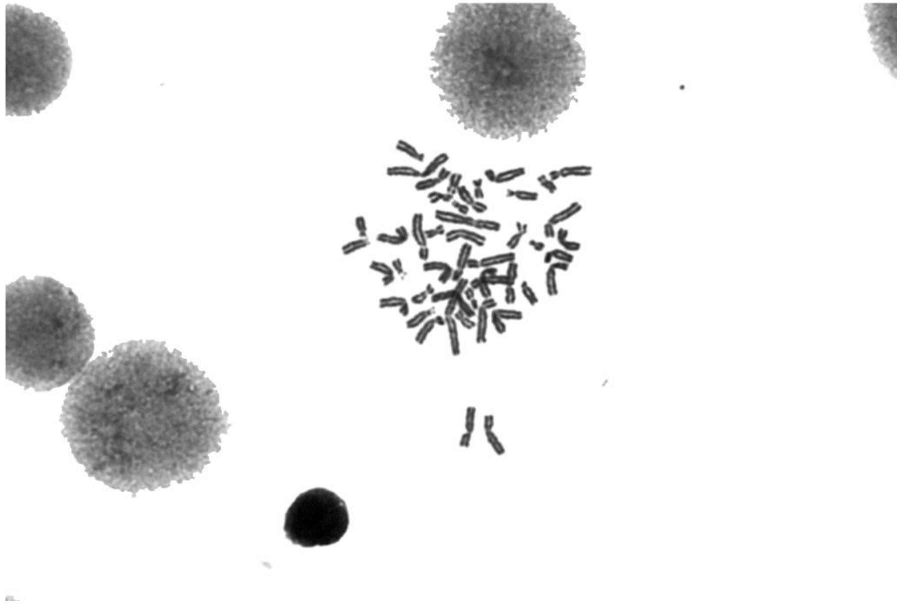

Karyotyping analysis reveals the presence of extrachromosomal DNA in the UACC-1598-4 cell line(A) (shown by an arrow), SKOV3(B)

Supplementary 2: The expression of Vimentin (marker for mesothelial cells) in UACC-1598-4.

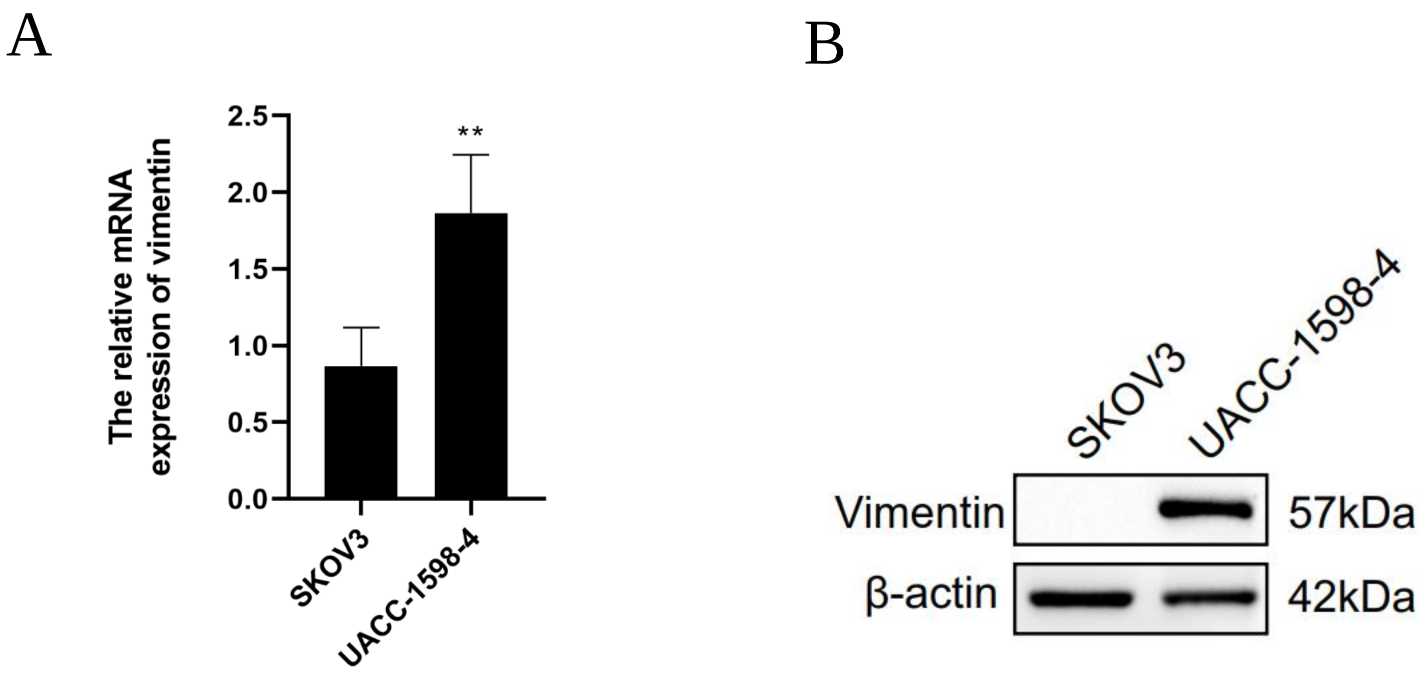

(A) The relative mRNA expression of Vimentin. (B) The protein level of Vimentin

Supplementary 3: Migration and invasion of UACC-1598-4 and SKOV3

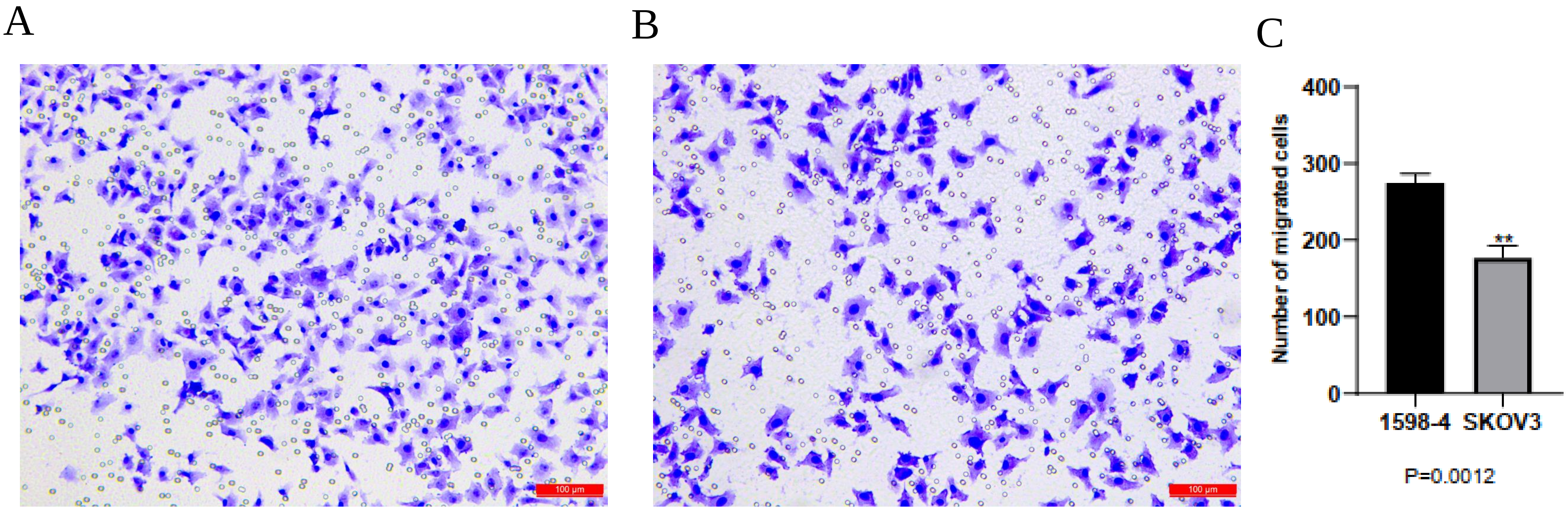

.(A) UACC-1598-4.(B) SKOV3.(C)Number of migrated cells of 1598-4 and SKOV3.

Supplementary 4:Validation of model prediction effectiveness in GEO dataset GSE72094

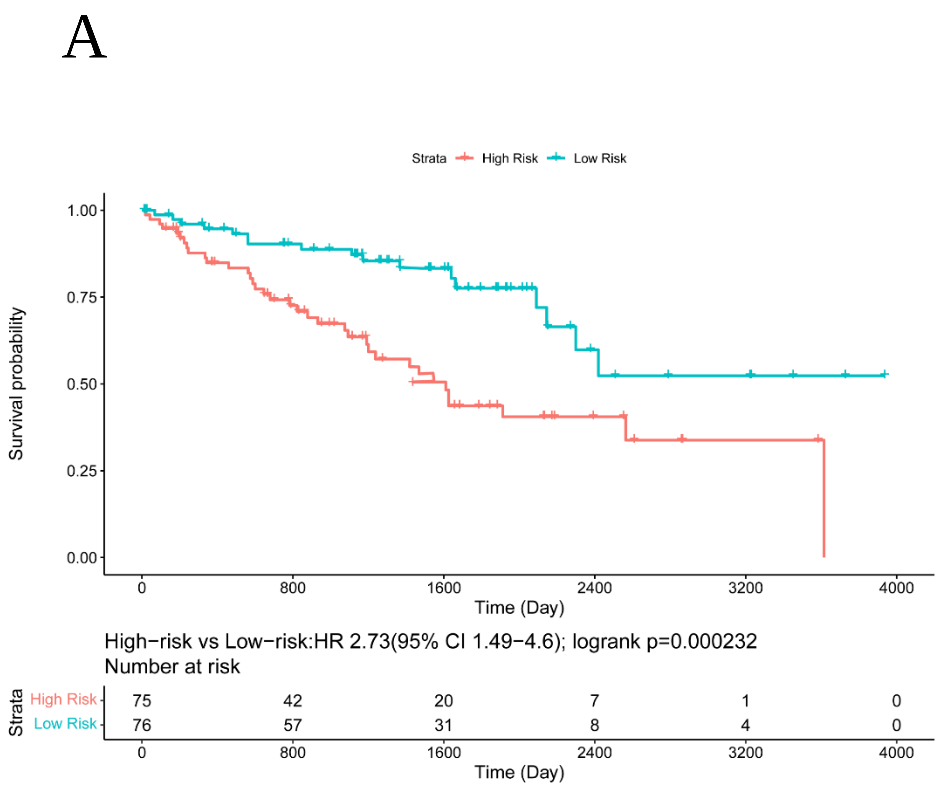

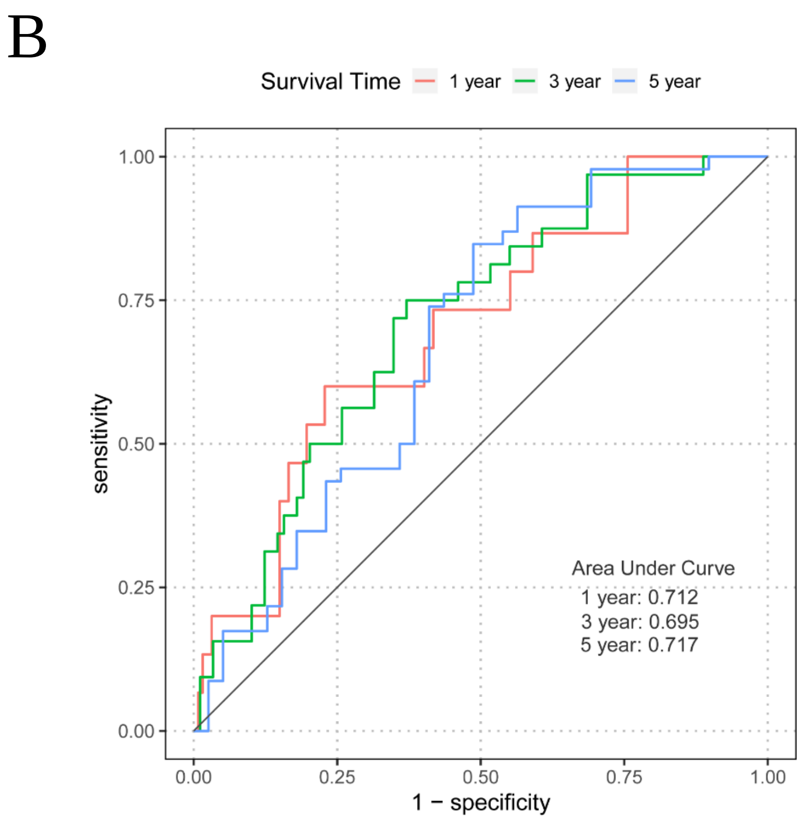

(A) Kaplan-Meier survival curve between high and low-risk groups.(B).Kaplan-Meier survival curve between high and low-risk groups.
