# Supplementary figures and images for: A novel extrachromosomal circular DNA related genes signature for overall survival prediction in patients with ovarian cancer
Source: BMC Med Genomics. 2023 Jun 19;16:140. doi: 10.1186/s12920-023-01576-x (PMC10278296; doi:10.1186/s12920-023-01576-x)

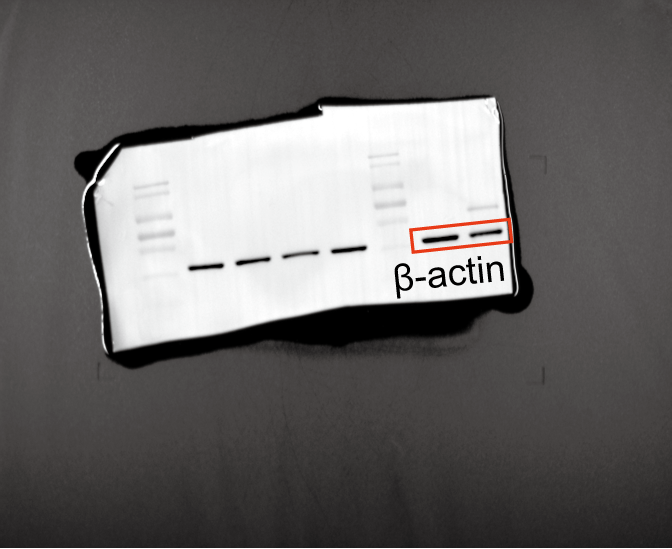

Supplement: Supplementary file 2 — Additional file 2. [file 12920_2023_1576_MOESM2_ESM.tif]

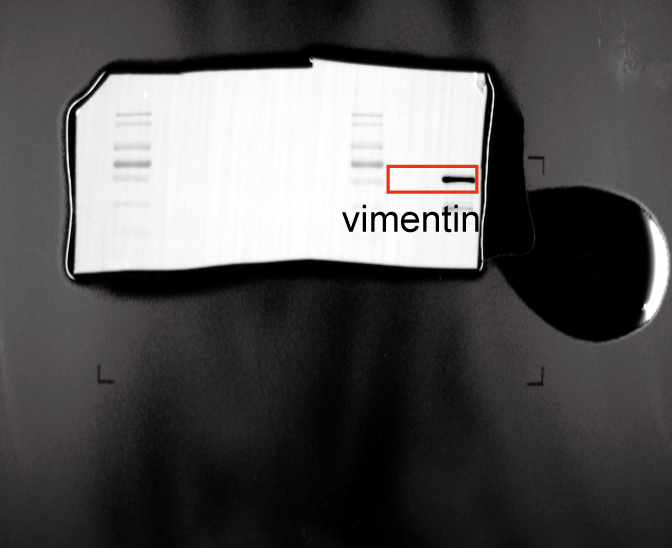

Supplement: Supplementary file 3 — Additional file 3. [file 12920_2023_1576_MOESM3_ESM.tif]
